# “Being prevented from providing good care: a conceptual analysis of moral stress among health care workers during the COVID-19 pandemic”

**DOI:** 10.1186/s12910-023-00993-y

**Published:** 2023-12-09

**Authors:** Martina E. Gustavsson, Johan von Schreeb, Filip K. Arnberg, Niklas Juth

**Affiliations:** 1 Global Disaster Medicine; Health Needs and Response. Department of Global Public Health, Karolinska Institutet, Tomtebodavägen 18, Stockholm, 171 77 Sweden; 2 Centre for Research ethics and Bioethics (CRB), Department of Public Health and Caring Sciences, Uppsala University, Uppsala, Sweden; 3 Stockholm Centre for Healthcare Ethics (CHE), Department of Learning, Informatics, Management and Ethics (LIME), Karolinska Institutet, Stockholm, Sweden; 4 National Centre for Disaster Psychiatry, Department of Medical Sciences, Uppsala University, Uppsala, Sweden

**Keywords:** Moral stress, Moral distress, Ethical/moral challenges, Health care workers, COVID-19 pandemic, Conceptual analysis

## Abstract

**Background:**

Health care workers (HCWs) are susceptible to moral stress and distress when they are faced with morally challenging situations where it is difficult to act in line with their moral standards. In times of crisis, such as disasters and pandemics, morally challenging situations are more frequent, due to the increased imbalance between patient needs and resources. However, the concepts of moral stress and distress vary and there is unclarity regarding the definitions used in the literature. This study aims to map and analyze the descriptions used by HCWs regarding morally challenging situations (moral stress) and refine a definition through conceptual analysis.

**Methods:**

Qualitative data were collected in a survey of 16,044 Swedish HCWs who attended a COVID-19 online course in autumn 2020. In total, 643 free-text answers with descriptions of moral stress were analyzed through content analysis.

**Results:**

Three themes emerged from the content analysis (1) “Seeing, but being prevented to act; feeling insufficient/inadequate and constrained in the profession,” (2) “Someone or something hindered me; organizational structures as an obstacle,” and (3) “The pandemic hindered us; pandemic-related obstacles.” The three themes correspond to the main theme, “Being prevented from providing good care.”

**Discussion:**

The main theme describes moral stress as various obstacles to providing good care to patients in need and acting upon empathic ability within the professional role. The themes are discussed in relation to established definitions of moral stress and are assessed through conceptual analysis. A definition of moral stress was refined, based on one of the established definitions.

**Conclusions:**

On the basis of the study results and conceptual analysis, it is argued that the presented definition fulfils certain conditions of adequacy. It is essential to frame the concept of moral stress, which has been defined in different ways in different disciplines, in order to know what we are talking about and move forward in developing prevention measures for the negative outcomes of this phenomenon.

**Supplementary Information:**

The online version contains supplementary material available at 10.1186/s12910-023-00993-y.

## Background

When confronted with morally challenging situations, in which it is difficult to act in line with their own moral values or professional ethical standards, health care workers (HCWs) may experience transient moral stress. However, moral distress, a lingering negative stress reaction, can ensue as a result of frequent, severe, and/or long-term morally challenging situations [1]. In times of crisis, such as disasters and pandemics, there is an increased risk of morally challenging situations due to a scarcity of resources, which creates an imbalance between patient needs and the capacity to meet them [2]. Despite limited time and resources, HCWs strive to provide high-quality care to patients in these situations [3]. In order to facilitate a better understanding of moral stress and related concepts, it is important to establish a clear definition of the concept. Also, conceptual clarity is needed to consistently measure the phenomenon in question and, moreover, aid in the development of effective organizational interventions and support for HCWs on how to manage moral stressors. Moral stress is not a clinical diagnosis, and it is distinct from other forms of stress reactions in that it is rooted in moral issues rather than other types of stressors [4, 5]. Through the establishment of a clear definition of moral stress, researchers and HCWs will be better equipped to identify and address the unique challenges faced in the health care setting, particularly in times of crisis.

The definitions of moral stress and moral distress vary and have evolved since the earliest definition was presented by Andrew Jameton: *“Moral distress arises when one knows the right thing to do, but institutional constraints make it nearly impossible to pursue the right course of action.”* [6, 7]. A broader definition was later developed by Kälvemark, Höglund and Hansson: *“Traditional negative stress symptoms that occur due to situations that involve an ethical dimension where the health care provider feel she/he is not able to preserve all interests at stake.*” [8]. However, a lack of consensus and clarity regarding this phenomenon remains in the literature. A review of moral stress from 2020 revealed that definitions and concepts still vary [9].

Another recently published study highlighted that the varied concepts become an obstacle to implementing effective measures to prevent and reduce the consequences of moral stress [10]. Hence, it is crucial to frame HCWs’ own descriptions of morally stressful situations during crises such as the COVID-19 pandemic, as that could provide support for development of the concept. The definition by Kälvemark derived from focus group discussions with HCWs, centered on their daily practices in normal health care. However, to our knowledge, the present study is one of the first conceptual analyses which involves HCWs’ own accounts of experienced specific morally stressful situations during a health crisis such as the COVID-19 pandemic. Conceptual analysis is often used to refine definitions and concepts and make a vague or multifaceted concept already in use more precise [11, 12]. Assessing the most commonly used definitions and reviewing those in relation to HCWs’ own descriptions of morally stressful situations can provide not only more clarity about the concepts used, but also serve as an assessment of the need of a further refined definition. For this study, the term moral stress is used overarchingly to include moral distress since there is no separation between moral stress and moral distress in the Swedish language (coupled to the question in which the free text answers were provided). Also, this study takes a stance in that moral distress is developed depending on the frequency, intensity, and duration of moral challenges. In that sense, it is the description of the morally stressful situation that is the focus in this study.

### Aim

This study aims to map and analyze Swedish HCWs’ descriptions of moral stress and develop a definition of moral stress through conceptual analysis.

## Methods

### Procedure

On commission from the Swedish National Board of Health and Welfare, Karolinska Institutet developed a web course related to COVID-19 for Swedish HCWs, including administrative and support staff [13]. In September 2020, 153,300 individuals participated in the course. Among these participants, those who defined themselves as HCWs were invited by email to participate in a web survey between September and October, 2020 [14]. See Supplementary file 1, Questionnaire guide, for the survey questions.

The survey was administered through a web-based, secure platform, the Research Electronic Data Capture (REDCap) tool, hosted by Karolinska Institutet [15, 16].

### Participants

The respondents came from all regions in Sweden. Due to a lack of data, it was not possible to calculate the share of eligible HCWs out of all 153,000 who received the survey. Among the 23,425 replies received, 6,551 were removed due to unfinished answers in the background information section. Furthermore, 832 duplicate entries were removed, which resulted in a final sample of 16,044 participants. Only those who responded “yes” to the initial question “to what extent have you been in such a situation [involving moral stress]?” were included (n = 8721), since only they were given the opportunity to provide a free text answer (below we further explain the conditions of exclusion). Most of the respondents were employed in a health care profession involving direct patient care, such as health care assistants, assistant nurses, nurses, and physicians. Managers and coordinators were professions in the minority. Professions related to patient care but not directly involved in COVID-19 care included e.g. dentists/dental nurses and radiologists. The majority of the respondents (85%) were females [14].

This study focuses on the qualitative analysis of specific parts of the survey related to moral stress, while quantitative analyses on moral stress and moral distress are reported elsewhere.

For this study, free-text answers in Swedish were collected and analyzed related to the second question (described in detail above) with the free-text response: “I have experienced moral stress in different type of situation, namely…” The reason for analyzing free-text answers in relation to this question only, is that the question deals with different definitions of moral stress. Thus, the number of free-text responses that are analyzed is equal to the number of questionnaires with free-text responses (there is one and only one free-text response that is analyzed). The number of free-text responses was 826. Of these, however, 183 were not analyzable due to only one point or the like, or those that did not relate to moral stress or lacked enough content to be analyzed, for example “urgent situation” or “difficult”.

### Survey questions

Questions related to moral stress were developed based on the results of a scoping review [1] and a qualitative study [17] that investigated characteristics of moral stress, moral distress, and its consequences among Swedish disaster health responders. The survey was piloted among four disaster-oriented health responders and refined. Thereafter, the survey questions were piloted a second time with four Swedish HCWs with experience from the current pandemic and refined again.

A description of moral stress (to provide clarity regarding the separation from other types of stress reactions) was presented to the participants introducing the section of questions related to moral stress: “Some situations may mean that you cannot follow and act in line with your moral values. These situations may give rise to moral stress, e.g., feelings of powerlessness, frustration, helplessness, and anger/sadness. The situations may, for example, be that you have needed to make decisions even though the options available seemed wrong, or where you have been prevented by circumstances from doing what is in line with your values, or where you have been involved in a decision that went against your beliefs due to another person’s actions or decisions.” Thereafter, the participants could respond, on a five-point Likert scale (response categories: *never, rarely, occasionally, often, very often*), to if they had “been in situations of moral stress.” Next, the participants rated their levels/the perceived severity of moral stress in five situations on a seven-point Likert scale: “There may be situations where you cannot do what you feel is morally right in your decisions or actions. Here, we ask you to rate the extent to which these situations have been stressful.” The alternatives were: (1) Ethical dilemma: when all the alternatives felt wrong, but I had to act/make a decision; (2) I made or was included in a decision that was not aligned with my moral values; (3) When other people’s decisions hindered me from acting in accordance with my moral values; (4) When other circumstances hindered me, such as lack of time or materials and structural resources; and (5) When I took action, but I felt that it was not sufficient based on my moral perceptions. After rating these five situations, participants could provide a free-text response to describe another situation: “I have experienced moral stress in a different type of situation, namely…”.

### Analysis

Content analysis was performed on the 643 responses, in which meaning units were generated from the free-text answers and given a code to describe the content [18]. The codes were then categorized into groups (243 subcategories) based on their contents. The subcategories were sorted into 10 different larger categories. These categories were subsequently grouped into three themes, which in turn were subsumed by a main theme. See Supplementary file 2 for an overview of the content analysis. When the content analysis was finalized, quotes were translated into English using DeepL. For accuracy, the translated parts have been reviewed by a translator knowledgeable in both Swedish and English. Within the quotes, square brackets denote additional inserted information, while double forward slash “//” denotes information that has been removed for clarity.

#### Conceptual analysis

The conceptual analysis is based on a model specifying a number of conditions of adequacy that a definition should fulfil [12]. These criteria can be fulfilled to various degrees. The criteria are: (1) The definition should be consistent with language use (language use requirement) and here empirical input is of use [11]. (2) The definition should be as precise as possible, to minimize doubt about which phenomena that can be included or not (precision requirement). (3) The definition should form the basis of an explanation as to why certain phenomena should be included or not (theory requirement). (4) The definition should allow for it to be empirically straightforward to determine whether a certain phenomenon should be included in the definition or not (reliability requirement). (5) The definition should be as simple and homogenous as possible and, consequently, there should be few exceptions or modifications (simplicity requirement). Lastly, (6) There should be a specific goal with the definition (target requirement). In this case, it is to allow for clarity and empirical measurability of moral stress [12]. Refining a concept like moral stress is done with the intention to ensure that the concept fulfils these criteria. It should be noted that these criteria may conflict with each other. For instance, precision and adherence to ordinary language use often conflict, as ordinary language use is generally vague and imprecise. In such cases, one must argue why prioritizing one criterion above another is appropriate in a certain context.

## Results

In the content analysis, three interlinked themes were identified which corresponded to the main theme “Being prevented from providing good care”. The three themes were: (1) “Seeing, but being prevented to act; feeling insufficient/inadequate and constrained in the profession,” (2) “Someone or something hindered me; organizational structures as an obstacle,” and (3) “The pandemic hindered us; pandemic-related obstacles.” The main theme describes moral stress as consisting of various obstacles to the provision of good care to patients in need and to acting based on empathic ability within the professional role.

### Theme 1 “Seeing, but being prevented to act; feeling insufficient/inadequate and constrained in the profession”

This theme involves three categories: (1) Not being taken seriously, (2) Feeling inadequate/insufficient, and (3) Acting outside one’s area of competence.

The first category, “*Not being taken seriously*,” consists of seven subcategories. It represents a frustration regarding being unable to address problems and not being heard, not being trusted in one’s professional judgment, not being valued, or not being heard by the manager/leader when highlighting problems. Further, observing inaction on the part of the employer or leadership even though a problem was obvious, and lacking any possibility to address political decisions.

*I heard about/witnessed situations that were contrary to my moral opinion, situations that I did not have the opportunity and/or resources to influence.*

*[I] raised risks regarding safety/spreading of infection, but wasn’t listened to, and our manager was initially on vacation and really didn’t understand the seriousness of the risks that we were being exposed to.*

The second category, “*Feeling inadequate/insufficient*,” consists of 43 subcategories and is the second most common source of moral stress seen in the responses. This category involves reports of doing everything you could, but it not being enough in various aspects – such as being hindered to give comfort and care to patients in need, witnessing increasing loneliness without being able to address it, having too little time to attend to patients and watching patients die even though you have done everything you can. Frustration was reported at being unable to be present for and give comfort to patients’ next of kin, and being unable to support one’s colleagues as needed. Moreover, there were reports of powerlessness among HCWs when contracting COVID-19 themselves, and powerlessness and a sense of inadequacy when non-COVID-19 patients were abandoned or given lower priority. It was reported as especially problematic when patients did not receive appropriate medical care and when there was a lack of opportunities for follow-up of patients.

*Patients felt very lonely and isolated, the fact that we, as staff, could only partly alleviate patients’ stress and anxiety, that we were not enough*.

*Feeling powerless at the ICU when people became real people that others have longed for, when a postcard, a letter, or a picture was pasted by the patient’s side // and when the outlook was bad, that they didn’t have someone beside them and that the little letter or greeting from a child or a father/mother, the nearest [and] dearest sending a greeting // that broke your heart, these people were missed… When next of kin could only come to say a last goodbye when the outlook was bad, when you stood outside the ICU and instructed next of kin on how to dress for the last farewell….*

The third category, “*Acting outside of one’s area of competence*,” represents 14 subcategories that relate to having lack of competence and knowledge in the professional role due to a new disease and transfer of HCWs to workplaces with a surge of patents during the pandemic. Moreover, the category covered challenges related to working with new staff who lacked the appropriate competence. Furthermore, being left alone with too many responsibilities, watching the care of patients deteriorate due to lack of competence and being thrown into new work roles without introduction or preparation were common descriptions of moral stress.

*Felt morally wrong to work without having the right experience, but had to do my best.*

*Support for workers on the floor, pressured co-workers to work with inexperienced colleagues to a higher extent than I normally do*.

*Had to work with patients from other wards where I felt that my competence was not sufficient and that resulted in me experiencing that the care we provided became worse which created moral stress.*

### Theme 2 “Someone or something hindered me; organizational structures as an obstacle”

The second theme consists of three categories: (1) Decision-making, (2) Teamwork, and (3) Information and communication by organizational management.

The first category, “*Decision-making*,” consists of 26 subcategories that describe difficulties related to one’s own decision-making, incorrect decisions being made by others, lack of decision-making, and lack of decisions by leadership. Being forced to make decisions based on too little medical information, making decision in line with moral values, but against protocols, being unable to make decisions, seeing decisions that need to be made but lacking authority, and feeling alone in decision-making and responsibility were described as challenges. Moreover, moral stress was reported when being forced to follow erroneous medical prescriptions or doctors’ decisions which were against one’s moral values, and being forced to adhere to others’ decisions despite knowing that they would prove ineffective. Lack of information and improper guidelines were perceived as leading to incorrect decisions. A lack of support in decision-making and experiencing that management did not make overall decisions, forcing frontline HCWs to make difficult prioritizations, were also described as problematic. Also, it was experienced as morally stressful to feel a duty to support one’s employer in relation to external parties when one did not agree with the employer’s decisions. Further sources of moral stress included situations when a patient decided what HCWs should do, or when a patient was dissatisfied with care. There were also difficulties in determining what is right when making decisions regarding patients with psychiatric conditions and dementia. Feeling alone with decision-making was reported by those in a leadership role.

*When I, in front of others, must stick to a position that I know will cause stress to those who will work based on that position, but at the same time we as an organization must observe regulations and guidelines*.

*I was trained in making decisions that go against my moral compass, and know that people probably died needlessly due to that.*

*Being forced to make decisions on uncertain grounds which could cost lives and affect my co-workers’ mental and physical health.*

The second category, “*Teamwork*,” consists of 15 subcategories that reflect situations related to collaboration in the workplace. Participants reported situations such as lack of initiative from team members, lack of communication between professions, lack of coordination leading to waste of personal protective equipment (PPE), and frustration among colleagues which made collaboration difficult. Furthermore, there were several examples of situations that created tensions in the teamwork: colleagues who behaved poorly with patients, colleagues who increased risks when not triaging right, colleagues who did not do their job, colleagues not observing guidelines, colleagues not providing collegial support, colleagues becoming paralyzed which increased the workload, colleagues’ fear of the disease, and colleagues who refused to work with COVID-19 patients.

Situations regarding management’s role related to putting too much pressure on staff and a lack of initiative from managers/leaders. Those in a leadership/managerial role reported issues regarding pressuring staff too much or pressuring them to work with new colleagues – things that they would not usually do.

*Rushing/hurrying and unresolved frustrations among other colleagues who should cooperate professionally.*

*Colleagues [staying] at home, [due to] fear of contracting the disease.*

The third category, “*Information and communication by organizational management*,” consists of 33 subcategories related to inconsistency in guidelines, poor overall management, and lack of collaboration and coordination between instances. There were many reports regarding poor leadership such as the manager not thinking PPE was needed, the employer making decisions against regulations, getting mixed messages from management, a lack of competence among management leading to decisions based on feelings rather than on evidence, management not being visible in the workplace, a lack of decisions and lack of responsibility on the part of management, a lack of coordination between managers, and a lack of support and initiatives from management. Regarding the inconsistency of guidelines, the responses were centered around new directives and guidelines that could not be adopted locally, a lack of clear guidelines, a lack of time to gather new knowledge and new information, and a lack of tutorials.

The participants in managerial positions described challenges related to leading and not working close to patients, and to implementing new ways of working for the staff. They also mentioned difficulties regarding planning and gathering staff amid all the concerns related to the pandemic, and difficulties in evaluating if staff were following the new procedures. Morally stressful situations regarding a lack of coordination and collaboration centered around a lack of collaboration between the state, the county councils, and the municipalities, a lack of collaboration between different wards, and differing routines between different care instances.

*That management did not make decisions on the direction, so that we on the floor had to make the difficult prioritizations.*

*Non-decisions, management has more or less avoided coming to the workplace altogether.*

*Management makes decisions that we on the ‘floor’ have to follow even though we know that they don’t work.*

### Theme 3 “The pandemic hindered us; pandemic-related obstacles”

The last theme consists of four categories: (1) Priority setting, (2) Lack of resources, (3) Infection prevention measures, and (4) Limitations regarding end-of-life care.

The first category, “*Priority setting*,” represents 10 subcategories. Issues were reported regarding balancing different needs against risks, balancing mental needs among psychiatric patients against infection prevention, difficulties in prioritizing some patients and abandoning others, and the decreased quality of care resulting from this prioritization. Frustrations related to too much administrative work were common. Reports regarding the managerial role were centered around challenges in dealing with the role and conflicts related to providing care without risking the health of staff. Further, managers reported difficulties in advocating the “next best care” due to a lack of resources.

*The stress increased because you didn’t have time for all your patients because it was extremely time-consuming to care for COVID-19 patients [and] at the same time [care for] patients who were not isolated.*

*Prioritization of COVID-19 patients made patient safety low.*

The second category, “*Lack of resources*,” consists of 34 subcategories regarding material resources, staff, and lack of time. Reports regarding a lack of materials or inadequacy of materials such as PPE, gloves, soap, and disinfectants were common – however, the patients still need to be cared for. There were also reports of a lack of medical equipment such as oxygen, ventilators, and equipment for sampling.

High needs combined with illness and stress among staff led to a lack of staff, which resulted in overtime hours, changes in schedules, and work placement – at times, even sick staff had to work. Furthermore, a lack of hospital beds due to a surge of patients led to some patients being prematurely discharged, being sent to other hospitals or care instances, or having to stay at home. It was common with responses regarding not having enough time to care for patients, beyond the most essential needs.

There were also responses that the situation during the peak of the pandemic was extraordinary and could be likened to war-time care. On the other hand, some participants mentioned that they found work meaningful during the pandemic, and that it was frustrating to return to a lack of resources when working under normal conditions.

*Being two night staff for 20 residents of whom 12 died in COVID-19.*

*For me, COVID care was the most well-functioning health care I’ve ever been part of – experiencing a lot of stress (and anger) at getting back to // “the line of people waiting for care” at my regular job, where resources, support, etc., are lacking. Unusually obvious that there is a human value, a decision, behind how patients will fare and what it should be like to work with them.*

*The care then and there was morally right, but if we had lifted out ONE patient and cared for them now, the care we had then would be wrong. We had to engage in war-time care, not the usual critical care we are used to.*

The third category, “*Infection prevention and control measures*,” consists of 45 subcategories and includes the most responses. It was common that the PPE felt like a barrier to giving comfort and sound care to patients. Further, it was reported as stressful to, as a HCW, be potentially contagious for patients. Those who worked with care of COVID-19 patients felt frustration at being unable to provide care to other patients due to the restrictions. Other respondents reported frustration at having to witness diminished social support and activity affecting patients’ wellbeing, as restrictions and isolation led to increased loneliness among patients, especially the elderly. Other common responses were challenges related to patients with positive COVID-19 not wanting to be isolated, forcibly isolating patients who did not understand why or protested, being unable to explain the reasons behind the restrictions to patients with conditions like dementia, and witnessing or being forced to give compulsory care to patients.

Visiting restrictions were another common source of moral stress, for instance being forced to serve as a guard and argue with next of kin not respecting restrictions, giving cancer diagnosis without next of kin being there, giving bad news over the phone, separating COVID-19-positive parents from their babies, partners not being allowed on the delivery ward, doctors not doing bedside assessments of patients, using only video calls for medical assessments, and avoiding certain treatment to patients due to the risk of virus transmission.

*When everything that these people include in quality of life has to be cancelled for reasons that they do not understand or that can be explained concretely or demonstrated, leaving them almost socially isolated with only staff around.*

*Protective equipment was scarce and management consistently stated that visors were sufficient for patient care. Experienced a lot of moral stress when having to send staff into an environment that I myself didn’t consider safe.*

The fourth category, “*Limitations regarding end-of-life care*,” represents 16 subcategories. Common responses included that palliative care was the only option for patients or that patients were wrongly classified as palliative and did not receive the right care. Further, there were many responses about unnecessary medical efforts being given to patients only for the sake of next of kin, doctors avoiding decisions on discontinuing life support measures, and feeling like they participated in prolonged suffering without having any means to influence. Witnessing loneliness during end-of-life care due to visiting restrictions was a common response, exemplified by turning down next of kin who wanted to meet their family members, talking to next of kin regarding end-of-life care and having to say that only a few of them could come to visit, and being unable to give comfort to next of kin. There were also responses related to frustrations regarding how dead patients were treated at the beginning of the pandemic.

*During the pandemic // some doctors thought it was better to make patients palliative, but we would sedate them so they could tolerate the treatment they were receiving until next of kin came. Even though most patients we cared for were lying there screaming ‘I don’t want to live. Let me die!’ and ripped off [Non-invasive ventilation] masks or [High flow nasal cannula]. We as the nurses/nursing assistant team had to give patients a lot of sedation // wrestle them into bed and forcible hold masks in place. Just to let the next of kin see that we had done everything for their next of kin. Was this a dignified death for the patient? NO.*

*Suddenly, according to staff at the residential homes, they were giving morphine and midazolam injections to elderly people in so-called palliative care who had tested positive for COVID-19 but were not particularly ill and thus actually gave them active euthanasia. How hard can it be to provide oxygen in residential care? That they choose to more or less kill people instead. There were many times we were told that the patient in question had been very alert just a few days before….*

## Discussion

This study analyzed HCWs’ own descriptions of morally stressful situations during the COVID-19 pandemic in Sweden. Content analysis of these descriptions resulted in three themes (1) “Seeing, but being prevented to act; feeling insufficient/inadequate and constrained in the profession,” (2) “Someone or something hindered me; organizational structures as an obstacle,” and (3) “The pandemic hindered us; pandemic-related obstacles”, which all related to the overarching theme: “Being prevented from providing good care.” In the following, we first discuss the conceptual analysis of the definitions of moral stress in light of the findings from the content analysis before discussing the findings of the content analysis in detail.

Here, we present the two definitions of moral stress again to review their conditions of adequacy and compare those to the results of the content analysis. Jameton’s definition is *“Moral distress arises when one knows the right thing to do, but institutional constraints make it nearly impossible to pursue the right course of action”* [6, 7]. To a certain extent, it meets some of the conditions of adequacy such as the precision requirement and the reliability requirement, as it is clear which situations can and cannot be included in the definition. However, moral stress is restricted to specific situations where external institutional constraints block an individual from pursuing the right course of action. The definition by Kälvemark et al. is *“traditional negative stress symptoms that occur due to situations that involve an ethical dimension where the health care provider feel she/he is not able to preserve all interests at stake*” [8]. This is more in line with some requirements of adequacy, as the definition is wider. It better fulfils the requirements of language use, since not all moral stress is about being unable to act due to institutional constraints (see below). Moreover, it also better fulfils the criteria of theory and direction, as it explicitly deals with situations which involve an ethical dimension, capturing a wider variety of morally stressful situations. Further, looking more closely at the expression in Jameton’s definition, “when one knows the right thing to do,” one can question whether that is always possible when facing an ethical challenge. Sometimes there is no clear “right thing” to do.

Looking at the responses in this study, most of them are in line with the definition of Kälvemark et al. However, the second theme in our study, “someone or something hindered me, organizational structures as an obstacle,” is most clearly related to the definition of Jameton. Furthermore, the theme “the pandemic hindered us” could be in line with Jameton’s definition, as infection prevention control measures such as visiting restrictions institutionally constrained the HCWs from acting in accordance with their moral values.

Still, there are several examples from the results of the present study demonstrating why the definition of Kälvemark et al. is to be preferred to Jameton’s in terms of the three conditions of adequacy mentioned: language use, theory, and direction. Reports of moral stress in the category “not being taken seriously” or “feeling inadequate” in the first theme cannot be included in external constraints, as these situations could happen regardless of if such constraints were present or not. Furthermore, the category “limitations regarding end-of-life care” does not necessarily relate to Jameton’s definition, as these situations can develop without institutional constraints. That category is therefore more closely related to the definition of Kälvemark et al.: a situation with an ethical dimension where the HCW feels they cannot satisfy all the interests at stake.

Teamwork and information and communication from management could be related to institutional constraints in Jameton’s definition, especially in situations where management did not provide information and resources to do the right thing. However, in this study, reports also concerned feeling complicit in wrongdoings. This could be viewed as falling outside the scope of Jameton’s definition, as it can occur without institutional constraints. Thus, it is more related to Kälvemark’s definition of being unable to satisfy all the interests at stake.

The feeling of being prevented or unable to satisfy all the interests at stake in situations with an ethical dimension (Kälvemark et al.) can be related to all the situations described in the result of this study. However, there were two types of situations where it is debatable if they can be included in this definition. Both were categories in the first theme, where the HCWs (1) felt inadequate/insufficient or (2) felt they were not taken seriously. The first can be related to “being unable to preserve all interests at stake.” The second type of situation, not being taken seriously and not being heard, can be debated to fall outside Kälvemark’s definition. It is related more to a frustration at not being involved in decision-making and being silenced when raising concerns and therefore being unable to address or affect encountered problems. In order to encompass these types of situations as well, the definition should be expanded to include feeling inadequate and powerless in resolving ethical issues, which could be viewed as beyond “preserving all interests at stake” and more in line with the reliability requirement. A suggestion for such a definition is: “*moral stress is the kind of stress that arises when confronted with a moral challenge, a situation in which it is difficult to resolve a moral problem and in which it is difficult to act, or feeling insufficient when you act, in accordance with your own moral values*.” However, if one considers oneself as among those whose interests are not preserved, this revised definition is not necessary. It should also be noted that moral distress may be affected by the frequency, severity, and/or duration of the moral challenge.

In conclusion, it is evident that moral stress can develop even if constraints are absent. In a systematic review, an issue was mentioned regarding the expressions of constraints related to moral stress. Austin et al. [19] developed a definition based on Jameton’s, mentioning both external and internal constraints as causing moral stress. However, in the systematic review, mentioning such constraints was highlighted as an issue, as the expression “internal constraints” may create a sense of individual responsibility in relation to feeling moral stress [9]. Indeed, internal constraints could be viewed as coupled with an individual’s coping ability and thus valuable, as long as it is not only interpreted as placing the blame of feeling moral stress on the individual, without attempts to address the obvious causes of morally challenging situations (e.g., re-allocation of resources).

### The role of the professional

Results from this study point to the moral foundation of the role as a HCW: providing good care to patients in need. When HCWs experience difficulties and obstacles in providing good care to patients, moral stress develops. Indeed, other types of work-related stress could also increase in situations with a high workload, but these are separated from moral stress since the latter involves not being able to act upon empathy and according to own moral values. However, work-related stress might decrease the capacity to deal with moral stress. Consequently, these difficulties and obstacles in providing good care seem to have different features depending on the professional role; being an assistant nurse with little authority to make medical decisions, being a doctor trying to provide right medical treatment even when resources are lacking, being a nurse trying to oversee all patient care needs despite limited time, or being a manager with responsibility for both patient care and staff. Differing views on ethical problems between professionals are mentioned in a systematic review as a source of frustration, as there can be differences in how various professionals perceive a situation [20]. Another study has highlighted the need for interprofessional learning in clinical ethics, as nursing students learn to focus primarily on caring and medical students learn to focus mainly on diagnosis and intervention [21].

### Sources of moral stress

The most commonly reported sources of moral stress in this study were related to feelings of being inadequate/insufficient and a lack of resources, which is in line with results from a quantitative study [14]. It seems as though difficulties in providing good care to patients can have different characteristics. During the pandemic, there were certain characteristics that were more evident such as visiting restrictions and infection prevention measures. Lack of resources and feeling inadequate/insufficient could also be related to the pandemic, as they were the result of higher needs and a resultant lack of resources and time to give good care to patients leading to feelings of insufficiency. However, there were some reports that the lack of resources and time existed even before the pandemic. This mirrors a cross-sectional study of North American ICU physicians, which found increased (56.9%) or similar (41.2%) moral stress scores during the pandemic and pre-pandemic [22].

### Moral stress in extreme situations

Difficult prioritizations and being unable to give care to all patients were sources of moral stress and referred to as something that was necessary at the time, but felt extreme in hindsight. During disasters and pandemics, when needs are elevated and resources are limited, it could be more evident to HCWs that there is a need to prioritize due to the circumstances and not related to their own capacity. The issue of heightened ethical challenges mirrors the results of a qualitative study about ethical challenges among Syrian HCWs during extreme violence [23]. It might be less common when circumstances are normal to experience difficulties in providing good care due to a lack of resources. However, many of the situations described in this study are common sources of moral stress also in daily health care practice, such as non-beneficial treatment and issues related to end-of-life care [24, 25]. But it seems like disasters and pandemics are circumstances that can result in more complicated morally stressful situations, which is essential knowledge in preparations for HCWs working in disaster-like situations. But, again, these preparations are likely to be useful in ordinary situations as well. For instance, lack of resources is more accentuated in disastrous situations (this is the very definition of disasters) but lack of resources is not unique to them.

### Methodological considerations

This study reflects only participating HCWs’ own descriptions of morally stressful situations during the pandemic, and we do not have these HCWs’ descriptions of such situations before the pandemic. Therefore, one should be mindful when extrapolating to routine circumstances in the absence of a large-scale stressor like the pandemic. Also, this study only mirrors Swedish HCWs descriptions of moral stress, and more work is needed to investigate and compare these to the views of HCWs from other settings. Further, we note that other methods, such as interviews, might have provided more in-depth data, as free-text fields in surveys entail some limitations. Although many responses were brief, some were lengthy. Overall, we deemed them to provide a broad picture of morally stressful situations. The response rate could be deemed low and might therefore decrease the validity. However, the option to provide free-text answers was on a voluntary basis. Indeed, 643 responses is a large number of reports from HCWs and although we did not use sampling methods to maximize coverage or ascertain saturation, we suggest that the data for the content analysis represent a variety of experiences from different HCW categories. The introduction of moral stress that was provided in relation to the moral stress section in the survey could have influenced the responses. However, for HCWs to be able to answer the following questions there was a need to frame the area of moral stress as separate from other types of stress. The free-text answers were provided in relation to the question “other type of situation”, which could have resulted in answers beyond the scope of existing definitions. However, surprisingly they did not, instead the free text answers were well aligned with the broader kind of already existing definitions in the literature, see above. We cannot know for certain why that is, but it seems to lend some support for the conceptual analysis in this article. Furthermore, even though the survey was piloted in two phases, we note that a full content validity of the survey questions before its use would have increased the validity. However, that was beyond the scope of this study and balanced with the issue of conducting research in the midst of an ongoing pandemic to investigate a current phenomenon. For future use of these survey questions, we recommend further work on validity of the instrument. Lastly, the translation process from Swedish to English might have affected the meaning conveyed by the quotes. This was addressed through back-translation by an independent translator to ensure consistency.

## Conclusion

On the basis of the results of the content analysis and conceptual analysis, it is argued that the presented definition in this study fulfils certain conditions of adequacy. Through assessing a definition in detail based on these criteria and in relation to the results of this study, it is evident that a refined definition of moral stress could be useful. This definition better satisfies three of the criteria: language use, theory, and target requirements. Furthermore, we present a suggestion of a definition which is in line with an already established definition and could be more useful, as it might be simpler to use: “*Moral stress is the kind of stress that arises when confronted with a moral challenge, a situation in which it is difficult to resolve a moral problem and in which it is difficult to act, or feeling insufficient when you act, in accordance with your own moral values.*” It is essential to frame the concept of moral stress, which has been defined in different ways in different disciplines, in order to know what we are talking about and move forward in developing prevention measures for the negative outcomes of this phenomenon.

### Electronic supplementary material

Below is the link to the electronic supplementary material.

Supplementary Material 1

Supplementary Material 2

## Data Availability

Relevant data will be available from the authors upon reasonable request.

## Abbreviations

HCW: Health care worker
COVID-19: Coronavirus disease 2019
PPE: Personal protective equipment
