# Supplementary material for: “Being prevented from providing good care: a conceptual analysis of moral stress among health care workers during the COVID-19 pandemic”
Source: BMC Med Ethics. 2023 Dec 9;24:110. doi: 10.1186/s12910-023-00993-y (PMC10710698; doi:10.1186/s12910-023-00993-y)
Supplement: Supplementary file 1 — Supplementary Material 1 [file 12910_2023_993_MOESM1_ESM.pdf]

Additional file 1. Survey questions

Background questions

This first part contains introductory questions about you and your work.

Email

____

Date

____
(YYYY-MM-DD)

Gender/sex?

- ◯ Woman
- ◯ Man
- ◯ Other

Age

____
(Number of years)

What kind of relationship do you live in today?

- ◯ Married/cohabiting/partnership/open rel.
- ◯ Single/widow/widower
- ◯ Single/divorced/separated
- ◯ Single

(Select one option)

Do you have own children?

- ◯ Yes
- ◯ No

(Select one option)

What it your highest level of education?

- ◯ Upper secondary school
- ◯ Adult level in upper s. school
- ◯ College or university
- ◯ Other

(Select one option)

Other namely:

____

What is your main work situation today?

- ◯ Fulltime
- ◯ Parttime
- ◯ Studying
- ◯ Parental leave
- ◯ Housewife/hus
- ◯ band
- ◯ Job applicant
- ◯ Sick leave fulltime
- ◯ Sick leave parttime
- ◯ Retired
- Other, namely:

(Select one option, use free text option if needed)

In months

____
(How long have you been on sick leave (write in months)?

In months	______________________________<br>(How long have you been on sick leave (write in months)?
Other, namely:	______________________________
Has there been periods in your life when you have been on sick leave or in other ways not been able to work/study or take care of your children?	◯ No<br>◯ Yes
Was it due to experiences from your work?	◯ No<br>◯ Yes<br>◯ Don´t know

Questions regarding your work

In which municipality do you work?	◯ Stockholm<br>◯ Uppsala<br>◯ Sörmland<br>◯ Östergötland<br>◯ Jönköping<br>◯ Kronoberg<br>◯ Kalmar<br>◯ Gotland<br>◯ Blekinge<br>◯ Skåne<br>◯ Halland<br>◯ Västra Götaland<br>◯ Värmland<br>◯ Örebro<br>◯ Västmanland<br>◯ Dalarna<br>◯ Gävleborg<br>◯ Västernorrland<br>◯ Jämtland, Härjedalen<br>◯ Västerbotten<br>◯ Norrbotten
Have you, by your workplace, been offered and participated in additional training, beyond the online training(s), in COVID-19 care?	◯ No<br>◯ Yes
In which professional role do you work?	☐ Nurse<br>☐ Specialist nurse/midwife<br>☐ Nursing assistant/personal assistant<br>☐ Coordinator/manager assistant<br>☐ Assistant nurse<br>☐ Doctor<br>☐ Psychologist/psych.therapist/counsellor<br>☐ Physiotherapeut/occ. therapist/dietician<br>☐ Speech therapist/audiologist<br>☐ Chiropraktor/naprapist<br>☐ Biomedical analytics/laboratory assistant<br>☐ Prescriptian/pharmacist/ass pharmacist<br>☐ X-ray nurse/hospital fysicist<br>☐ Dental nurse/dentist/dental hygienist<br>☐ Head of operations/manager<br>☐ Other<br>(Please tick one or more options, based on what you have worked the most if you have had differing roles).
Other:	______________________________

In which type of inpatient care do you work?	☐ Intensive care<br>☐ Ward care (including infection ward)<br>☐ Specific COVID-ward<br>☐ Intermediate care<br>☐ Rehabilitation<br>☐ Geriatrics<br>☐ Palliative care<br>☐ Psychiatry<br>☐ Radiology dep.<br>☐ Emergency dep.<br>(Tick one or more options, use free text alternative below if needed)
Do you work within outpatient care? In that case, mark which type of outpatient care.	☐ Primary health care (including private general practice and local emergency reception)<br>☐ Outpatient clinic<br>☐ Outpatient psychiatry<br>☐ Rehabilitation<br>☐ Laboratory reception, phys.lab<br>(Tick one or more options, use free text alternative below if needed)
In which type of care, which are not mentioned above under hospital or open care, do you work? Free text-alternative are shown below.	☐ Prehospital emergency care (including ambulance care)<br>☐ Municipal care for the elderly (SÄBO, short term care, including home care)<br>☐ Municipal care (disability care, personal assistance)<br>☐ Home care (including advanced health care in the home)<br>(Tick one or more options, use free text alternative below if needed)
Other type of care, namely:	____________________<br>(Other type of care, namely)
How long time (in total) have you been working at your current workplace?	____________________<br>(Number of months)
For how long time have you been working in your current professional role?	____________________<br>(Number of months)
All HCW do not work with direct COVID-care but are affected by the circumstances. Mark the alternative which matches where you have worked.	◯ I have only worked with COVID-care<br>◯ I have only worked with non-COVID care<br>◯ I have worked with both types of care<br>(Choose one option)

For how long time have you then worked with COVID-care? Write in number of weeks.

(If you have marked only COVID-care or both, please note during for how long time? (number of weeks)

____

Moral Stress

In this first part, the questions are about your experiences of difficult situations in your professional role in your work during the COVID-pandemic.

Difficult situations and choices at work

On this page, the questions are about your experience of difficult situations in your professional role during the COVID pandemic. The following questions are about dealing with difficult situations and their consequences.

Some situations may mean that you cannot follow, and act on your moral values. These situations may give rise to moral stress, e.g. feelings such as powerlessness, frustration, helplessness, anger/sadness. The situations may be, for example, that you have needed to make decisions even though the options available to you seemed wrong, or where you have been prevented by circumstances from doing what is in line with your values, or where you have been involved in a decision against your beliefs, by another's action or decision.

To what extent do you feel that you have been involved in such situations?

- ◯ Never
- ◯ Occasionally
- ◯ Sometimes
- ◯ Often
- ◯ Very often

There may be situations where you cannot follow what you feel is morally right in your decisions or actions. Here we ask you to rate the extent to which these situations have been stressful.

For each option, indicate a level of your perceived moral stress (powerlessness/frustration). The scale is from 1-7 where 1 in none and 7 is extremely high. Mark one option per line.

I experienced moral stress in situations where...

	1	2	3	4	5	6	7
...all the options felt wrong, but I was still forced to make a decision	◯	◯	◯	◯	◯	◯	◯
...I took or was involved in making decisions that were not consistent with my moral values	◯	◯	◯	◯	◯	◯	◯
...other people's decisions prevented me from doing what was in line with my moral values	◯	◯	◯	◯	◯	◯	◯
...other circumstances prevented me, such as lack of time or material and structural resources	◯	◯	◯	◯	◯	◯	◯
...I acted but experienced it not "enough" according to my moral values	◯	◯	◯	◯	◯	◯	◯

A different kind of situation, namely: ______________________________

Grade what you have entered under "other type of situation":

◯ 1
◯ 2
◯ 3
◯ 4
◯ 5
◯ 6
◯ 7

(The moral stress I experienced in the following sitution was.
(1=none) (7=extremely high)

Difficult situations and choices at work

What was the causes behind the stressful situations?
Here we ask you to rate, overall, to what extent you think the following options contributed to the morally stressful situations.
Rate one option per line, 1 - 7, where 1 is not agreeing, 7 is fully agree.

	1	2	3	4	5	6	7
Resource scarcity e.g. lack of protective equipment, material, staff, care places	◯	◯	◯	◯	◯	◯	◯
Clash with the workplace/other actor's agenda and my morals	◯	◯	◯	◯	◯	◯	◯
Being forced to act outside my area of expertise	◯	◯	◯	◯	◯	◯	◯
Cultural, social & political aspects	◯	◯	◯	◯	◯	◯	◯
Leadership, structures within the team	◯	◯	◯	◯	◯	◯	◯
Conduct and decisions of colleagues	◯	◯	◯	◯	◯	◯	◯
Own safety over care of patients	◯	◯	◯	◯	◯	◯	◯
Isolation of patients which prevents visits and involvement by friends & family	◯	◯	◯	◯	◯	◯	◯
Difficulties for relatives to attend at end of life-care	◯	◯	◯	◯	◯	◯	◯

Other namely:

____
(This contributed to the stressful situation)

Please grade what you have entered under ”other”:

◯ 1
◯ 2
◯ 3
◯ 4
◯ 5
◯ 6
◯ 7

(This contributed to the stressfulness of the situation: rate according to (1=not true) (7=very true)

Support in dealing with difficult/stressful situations

What support was there for you to deal with the stressful situations? When you think about these situations overall, we ask you to rate how well the different alternatives correlate to you. The scale is from 1-7, where 1 is not true and 7 is completely true.

	1	2	3	4	5	6	7
It was up to me to solve the situation, find own support and come up with own solutions/ideas	◯	◯	◯	◯	◯	◯	◯
There was formal support (e.g. workplace support, End-of-shift talks, reflection groups)	◯	◯	◯	◯	◯	◯	◯
There was support in the form of psychologist/ counsellor, occupational health care, leadership support	◯	◯	◯	◯	◯	◯	◯
There was informal support (e.g. colleagues in the team, contact with friends/family/ other colleagues)	◯	◯	◯	◯	◯	◯	◯

There were other things, namely:

____
(There were other things, namely:)

Please rate how what you have written, was valuable for dealing with the stressful situations

◯ 1
◯ 2
◯ 3
◯ 4
◯ 5
◯ 6
◯ 7

(Rate how what you wrote was useful in dealing with the stressful situations, according to (1=not true) (7=very true))
