# Supplementary material for: “Being prevented from providing good care: a conceptual analysis of moral stress among health care workers during the COVID-19 pandemic”
Source: BMC Med Ethics. 2023 Dec 9;24:110. doi: 10.1186/s12910-023-00993-y (PMC10710698; doi:10.1186/s12910-023-00993-y)
Supplement: Supplementary file 2 — Supplementary Material 2 [file 12910_2023_993_MOESM2_ESM.pdf]

Additional file 2. Table of content analysis

| Subcategories | Categories | Themes | Main theme |
|---|---|---|---|
| | **1. Not being taken seriously** | **1. Seeing, but being prevented to act; feeling insufficient/inadequate and constrained in the profession** | **"Being prevented from providing good care."** |
| Frustration at not being heard | | | |
| Not being taken seriously | | | |
| Not being trusted in professional judgment | | | |
| Highlighted problems but management did not see/hear | | | |
| Inaction from management despite clarifying problems | | | |
| No possibility to address political decisions | | | |
| Inconsistent directives, ignorance, not being listened to, not trusted | | | |
| | | | |
| Tried and acted according to experience but it was not enough | **2. Feeling inadequate/insufficient** | | |
| Tried to provide good care but felt insufficient | | | |
| Felt powerless in certain situations | | | |
| Powerlessness when being ill yourself | | | |
| Dissatisfied patients | | | |
| Unreasonable demands at work that were never met | | | |
| Doing all you can, but the patient dies anyway | | | |
| Colleagues felt unwell but could not support them | | | |
| Cannot treat patients aged above 70 years | | | |
| That patients did not receive appropriate care | | | |
| Not being able to provide dental care to elderly who needed it | | | |
| Not being allowed to help even though you can | | | |
| Not being able to provide rehab to patients in risk groups | | | |
| Energy was not sufficient to cope with certain situations | | | |
| Not being able to follow up patients the way you should due to avoiding too many visits | | | |
| The lack of physical proximity when patients asked for it felt difficult | | | |
| Users felt lonely and isolated, could only partially help | | | |
| Being there for other patients | | | |
| Inadequacy during difficult conversations, a challenge to do it at a distance | | | |
| Not being physically present for next of kin, ill patients and dying patients | | | |
| Essential care was deprioritized by others or by me as other things needed to be done, leaving me with a feeling of unfinished work | | | |
| Not being able to sit with a dying patient | | | |
| Preventing next of kin from meeting their next of kin and not being able to hug or touch them when they showed worry and sadness | | | |
| Important instances were inaccessible and basic care of chronic patients was put on hold | | | |
| Witnessed situations that went against my moral conviction, where I did not have the means or resources to have an influence | | | |
| Not enough time or resources to provide individual care, unable to meet needs | | | |
| Lack of time, many people were lonely during the pandemic and needed to talk to someone | | | |
| Forced to work with COVID care and it was immoral to pause the care of other patients | | | |
| No possibility for follow-up of care which was given or planned | | | |

| | | | |
|---|---|---|---|
| Doing investigations/reports for the Social Insurance Agency [Försäkringskassan] for non-patients | | | |
| Acted as a doorman instead of writing charts and doing other work which had to be done later | | | |
| It was frustrating not being able to accept ordinary patients to help them with their legitimate but non-life-threatening situations | | | |
| Was trained in making decisions against my moral compass and know that patients probably died needlessly due to that | | | |
| No time to recover or reflect on things that you quickly had to act upon | | | |
| So many patients died | | | |
| Poor working environment with a lot of stress | | | |
| The care of parents where their child did not survive | | | |
| Video assessment of patients with breathing difficulties | | | |
| Sending home patients with a runny nose due to COVID-19 | | | |
| Patients within elderly care could not get an individual assessment by a doctor | | | |
| The staff that I am a manager for felt guilty in that they could work as usual | | | |
| Lack of time to care for the elderly and especially to listen to them | | | |
| The time pressure was as bad as always | | | |
| | | | |
| Very sick patients, a lot of solitary work without help and that you were not competent to do | **3. Acting outside one's area of competence** | | |
| Relocations of staff | | | |
| Thrown into a new workplace without introduction | | | |
| Managing work that you are not used to | | | |
| Forced to work in an area where you are not competent | | | |
| Lack of knowledge among other staff members | | | |
| Left alone with work that you cannot do | | | |
| Take care of ICU patients without the right competence | | | |
| Lack of competence led to lower quality of care | | | |
| Worked without experience but was forced to do my best anyway | | | |
| Uncertainty due to lack of knowledge | | | |
| Not being able to follow routines due to not having a mandate | | | |
| Receiving patients with COVID in radiology | | | |
| Relocated to nursing home but lacked competence in working with elderly | | | |
| | | | |
| Lack and unclarity of routines led to erroneous decisions | **1. Decision-making** | **2. ”Someone or something hindered me”; organizational structures as an obstacle** | |
| Could not limit risk of transmission due to insufficient decisions | | | |
| According to me, others made incorrect decisions without listening | | | |
| Premature discharges of patients from inpatient care | | | |
| Management making decisions that must be followed even though they do not work | | | |

| | | | |
|---|---|---|---|
| Defending the employer externally even though you do not agree with them | | | |
| Carrying out the manager's decision which you feel is inhumane in relation to the patient | | | |
| Following others' decisions despite knowing it will not work in the long run | | | |
| Doctor decisions which went against my values | | | |
| Changed medical guidelines for oncology care | | | |
| Had to make morally right decisions which were against routines | | | |
| Deciding whether ill staff members should work | | | |
| Forced to made decisions on uncertain grounds which could cost lives and affect my co-workers' mental and physical health | | | |
| Feeling lonely in responsibility and decisions | | | |
| Seeing decisions that must be made but not being included in decision-making | | | |
| As manager, you felt alone with your decisions | | | |
| Lack of support in decision-making | | | |
| Could not make any decisions on my own | | | |
| Forced to make medical decisions without knowledge | | | |
| Forced to follow substandard drug prescriptions | | | |
| Forced to pursue a course of action despite knowing it will cause stress for colleagues, but having to observe recommendations | | | |
| Management did not make any overall decision, so we at the frontline were left with the difficult prioritizations | | | |
| The patient decided what we should do | | | |
| Not related to COVID work, but it is difficult with care of dementia patients regarding moral stress and what is right | | | |
| Needing to request payment from patients with poor finances | | | |
| Lack of certificate for compulsory care within psychiatric care leading to psychosis | | | |
| | | | |
| | **2. Teamwork** | | |
| Poor communication between professions | | | |
| Colleagues who behaved poorly with patients | | | |
| Co-workers providing poor support | | | |
| Lack of initiative from co-workers and the manager | | | |
| Frustration among colleagues which makes collaboration difficult | | | |
| The managers put too much pressure on the staff | | | |
| Colleagues who do not do their job | | | |
| I pressured co-workers to work with inexperienced colleagues, which I normally do not stand for | | | |
| Poor coordination by doctors led to wasting of PPE | | | |
| Dangerous co-workers who do not perform triage well and thus put their colleagues in a dangerous position | | | |
| New colleagues became paralyzed, had to work more and longer to compensate | | | |
| Witnessing maltreatment without documentation | | | |
| | | | |

| | | | |
|---|---|---|---|
| Stressed by co-workers' fear of COVID-19 | | | |
| When you witness co-workers not acting in accordance with routines despite nudging | | | |
| Problems in the team when staff refused to work with COVID-19 patients | | | |
| | | | |
| Manager did not think PPE was needed | **3. Information and communication by organizational management** | | |
| Poor collaboration between the state and outpatient care | | | |
| Mixed messages from management | | | |
| New directives and guidelines which are not locally adoptable | | | |
| Insufficient decisions from authorities | | | |
| Discussions in the team due to it not being easy to grasp information despite clear directives and training | | | |
| Lack of knowledge among management led to decisions based on feelings, not evidence | | | |
| Unclear directives | | | |
| Non-decisions, management has almost avoided coming to the workplace | | | |
| Employer making decisions which are against the rules/regulations | | | |
| Responsible for communicating information | | | |
| Lack of information regarding guidelines and routines | | | |
| Lack of knowledge and time to gain new knowledge | | | |
| Unsatisfying communication or a complete lack of guidance | | | |
| Waiting for directives and the action plan not being in line with science/research results | | | |
| Care instances acting differently | | | |
| Insufficient supervision | | | |
| Closest manager provided poor support | | | |
| Lack of initiative from the manager | | | |
| The managers did not coordinate their actions | | | |
| Lack of leadership taking responsibility | | | |
| Managers' disinterest for patients/users | | | |
| A challenge to lead a hospital, not working at the frontline, to change ways of working at the wards | | | |
| Rapid changes in routines which had not been adopted at all instances | | | |
| Responsible for how things are planned and performed, information from different authorities has varied | | | |
| Unclear management | | | |
| Regulations about where to seek care, health advisors [Vårdguiden] have often sent patients to us, which we have no control over | | | |
| Difficult to assess whether the staff acted in line with new decisions | | | |
| Lack of knowledge within the disaster management (Katastrofledningsnämnden) led to decisions based on feelings and not evidence | | | |
| A collision between county, primary care and residential housing, a lack of communication between instances | | | |
| Collaboration with other wards, difficult to plan for a pandemic and gather everyone amid all the concern | | | |
| Felt like responsible personnel had underestimated the situation | | | |

| | | | |
|---|---|---|---|
| Other authorities'/instances' decisions have affected safety | | | |
| | | | |
| Balancing different needs and risks | **1. Priority setting** | **3. "The pandemic hindered us"; pandemic-related obstacles** | |
| Too much administrative work | | | |
| Prioritizing patients and refusing to give care which we would otherwise give was difficult | | | |
| A challenge regarding leadership, a conflict to provide care without risking the health of the personnel | | | |
| Prioritization of COVID patients over our patients resulted in diminished patient safety at the dialysis ward | | | |
| Difficult prioritization when several things happened at once | | | |
| Prioritizing between patients who were waiting for surgery | | | |
| Routines were prepared for various situations, but initially difficult to promote the next best thing due to lack of materials | | | |
| Choosing between mental wellbeing and the risk of infection for my client | | | |
| Could not book patients above 70 years for examination | | | |
| | | | |
| Lack of PPE and other materials such as hand disinfection and gloves | **2. Lack of resources** | | |
| Lack of staff | | | |
| Lack of ventilators | | | |
| There was no oxygen to give to the residents | | | |
| Substandard PPE, had to organize things ourselves | | | |
| Lack of co-workers due to sick leave and care of children | | | |
| Work with colleagues who are new and lack education (such as care assistants) | | | |
| Lack of staff and management cut down on staff/made savings to increase profit this year. Even though we protested and said that we are already on minimum staffing and need to prepare for the pandemic | | | |
| Promised next of kin that we could give good care at home, but we did not even have access to oxygen | | | |
| Lack of staff and overcrowding of patients are the two biggest problems in clinical medicine regardless of the COVID-19 situation | | | |
| Have been forced to work above the regulated maximum hours due to co-workers' sickness or stress | | | |
| Powerlessness regarding changes of schedules and being ordered to work (recovery time) | | | |
| In hindsight, I felt a bit too effective in emptying my ward to accept new patients | | | |
| The care then and there was right, but would not be acceptable now, we performed war-time care, which we are not used to | | | |
| Requesting staff to work overtime | | | |
| Lack of adequate sampling at the start [of the pandemic] | | | |
| Meaningfulness during COVID-19 work, but frustration regarding lack of resources in ordinary care/work | | | |
| Ill staff members were allowed to work | | | |

| | | | |
|---|---|---|---|
| Patients with suspected COVID-19 need monitoring, difficult with PPE. A need for someone to be with the patient all the time but resources were lacking | | | |
| Sampling without proper PPE (were lacking at the workplace) | | | |
| Was pregnant and supposed to avoid COVID patients, but this changed when more people became infected | | | |
| Discharged without being confirmed COVID negative | | | |
| When certain co-workers stayed at home due only to fear of contracting the disease | | | |
| Not allowing staff to use PPE even though they worked close to patients, e.g., for taking samples. The patients were not COVID-19 patients, but you never know, especially as most of our patients use intravenous drugs | | | |
| Quick discharge from hospital to nursing home which counteracted next of kin's involvement | | | |
| Sending patients to another hospital due to space restrictions | | | |
| Patients had to stay home due to space restrictions | | | |
| The staff did not have time, they isolated those who could, contact mostly for food and toilet visits | | | |
| Letting staff take sick leave "just in case," which led to overtime and high workloads for others | | | |
| Working despite a lack of PPE and staff | | | |
| Had to re-use PPE several times | | | |
| A lack of gloves, soap, paper and hand disinfection | | | |
| We did not receive any PPE, bought it myself but was told not to use it, which I did anyway | | | |
| Transporting elderly patients to hospitals far away | | | |
| | **3. Infection prevention measures** | | |
| Visiting restrictions for next of kin to severely sick patients | | | |
| Limitation of activities | | | |
| Met worried and frustrated next of kin due to visiting restrictions, this took time from patient care | | | |
| PPE feels like a barrier between caregiver and patient | | | |
| Isolation of patient with high risk of falling | | | |
| Cancellation of visiting restrictions leading to increased risk of transmission | | | |
| Gave cancer diagnosis without next of kin being present | | | |
| Bad news could only be communicated by phone | | | |
| Next of kin not respecting visiting restrictions | | | |
| Doctors never performed "bedside" individual assessments | | | |
| Isolation preventing social contacts | | | |
| Difficult to explain restrictions to dementia patients | | | |
| No partners could be present during delivery [delivery ward] | | | |
| Forcibly isolating a patient in a room | | | |
| Worried about spreading the disease to severely ill patients | | | |

| | | | |
|---|---|---|---|
| In prehospital care: only performing compressions in cases of cardiac arrest due to risk of transmission | | | |
| Patient who cannot receive certain treatment due to the staff's risk of contracting the disease | | | |
| Not being able to do medical assessment through video calls, could not meet patients due to risk of infection | | | |
| Got infected at work and transmitted the disease to next of kin, felt guilty and worried | | | |
| Hard to find time to change clothes between all the sick patients | | | |
| Patients who refused isolation despite positive COVID-19 | | | |
| Making the situation understandable to a dementia patient, why they cannot do this or that | | | |
| Restraining dementia patients | | | |
| Forcibly isolate patients who do not understand why | | | |
| Compulsory care | | | |
| Denying care to patients and assessment by the doctor even though this would usually have been offered | | | |
| Supposed to wear PPE even though that does not work with autistic children | | | |
| The daily work with patients, such as motivating talks and moral support, has been set aside due to all the restrictions | | | |
| Hard to make decisions regarding limiting a person's life to protect them against a virus when the person does not understand what a "virus" is or why daily life has to change | | | |
| Omitted care to newborns due to a cold and not being allowed to make home visits | | | |
| Spending time on changing clothes before approaching a cardiac arrest or critical patient | | | |
| Being a risk of infection to the most fragile patients | | | |
| Working with more distance and not having time due to restrictions and isolations, the patients' mental health has worsened | | | |
| Healthy people had to stay in their rooms to avoid risk of transmission | | | |
| Have to put up with crying, screaming, and bad words from next of kin | | | |
| Not being able to perform certain clinical assessment through video calls as we could not meet infected patients at the health care center because they were triaged to a so-called infection hub [infektionsnod] | | | |
| Those of us who work in outpatient care have not been allowed to use PPE. Patients should be viewed as healthy, as should we, when we are here | | | |
| Working at two clinics, was prevented from going to the other one as we should not mix personnel. I could not help these patients | | | |
| When my co-workers provide patient care without PPE even though a patient has suspected COVID-19, and when there are not enough scrubs for the extra staff. | | | |
| Residents in a nursing home could not dine together even though they could keep physical distance. Social distancing from co-residents created loneliness | | | |

| | | | |
|---|---|---|---|
| Consequences of visiting restrictions for patients who need support from next of kin in practically and mentally difficult situations | | | |
| That residents could not get critical care at the hospital | | | |
| Working in home environment, difficult to manage PPE and waste | | | |
| Not being able to participate in the care and supervision of personnel due to risk of infection | | | |
| Separation of COVID-19-positive parent from diseased newborn (non-COVID) | | | |
| | | | |
| Patients were wrongly classified as palliative and did not receive help | **4. Limitations regarding end-of-life care** | | |
| Palliative care without the patient being aware | | | |
| Only palliative care could be given | | | |
| Loneliness in end-of-life care | | | |
| Too much medical efforts for the patient, only for the sake of the family | | | |
| How the deceased were treated at the beginning of the pandemic. This changed and became more humane | | | |
| Doctors do not dare make decisions regarding cessation of life support | | | |
| That elderly people were not allowed to go to hospital but should receive palliative care instead | | | |
| Participating in prolonged suffering despite trying to influence this | | | |
| Providing palliative care in cases where it otherwise would not be given. The patient had been alert days before, but palliative care was ordered as oxygen was not available | | | |
| Consequences of visiting restrictions in palliative care, counteracting the individual's right to have someone beside them in their last hours | | | |
| Did not work as a team. The doctors promoted palliative care until next of kin came and saw that we "did everything we could." The doctors ignored the patient's best interests | | | |
| Conversations with next of kin in end-of-life care, which only a few of them could attend | | | |
| Saying no to next of kin who want to be with the dying patient | | | |
| When next of kin could not accompany a patient to the hospital even though the patient would probably die of their condition | | | |
| Understaffing of nurses resulted in long waiting times for adequate end-of-life care | | | |
